# Successful bronchial artery embolization using hydrogel coils for hemoptysis during extracorporeal membrane oxygenation

**DOI:** 10.1016/j.radcr.2022.07.025

**Published:** 2022-08-01

**Authors:** Takashi Nishihara, Yutaro Okamoto, Hideo Ishikawa, Naoki Omachi, Yoshiaki Yoshikawa, Kenichiro Ishida, Masayasu Toratani, Mitsuo Ohnishi

**Affiliations:** a Hemoptysis and Pulmonary-Circulation Center, Eishinkai Kishiwada Rehabilitation Hospital, 2-8-10 Kamimatsu-cho, Kishiwada City, Osaka 596-0827, Japan; b Department of Acute Medicine and Critical Care Medical Center, Osaka National Hospital, National Hospital Organization, 2-1-14 Houenzaka, Chuo-Ku, Osaka City, Osaka 540-0006, Japan; c Department of Radiology, Osaka National Hospital, National Hospital Organization, 2-1-14 Houenzaka, Chuo-Ku, Osaka City, Osaka 540-0006, Japan

**Keywords:** Bronchial artery embolization, Extracorporeal membrane oxygenation, Hemoptysis, Hydrogel coil

## Abstract

A 58-year-old woman with bronchiectasis presented with massive hemoptysis and severe respiratory failure, which required long-term extracorporeal membrane oxygenation with continuous heparin infusion. Bronchial artery embolization using hydrogel coils, which provide a greater volume occlusion than bare platinum coils, was performed; hemoptysis stopped and she fully recovered. No recanalization was observed on follow-up computed tomography angiography 2 months postbronchial artery embolization, and there had been no recurrence of bleeding at the time of this report (at least 6 months). Although continuous anticoagulation during extracorporeal membrane oxygenation might hinder complete vessel occlusion by metallic coils or induce early recanalization (because the homeostatic mechanism of coils depends on the patient's coagulability), our experience showed that bronchial artery embolization using hydrogel coils was effective and safe. Additionally, this case presents a successful example of anticoagulation management for patients with hemoptysis on extracorporeal membrane oxygenation who undergo bronchial artery embolization using coils.

## Introduction

Metallic coils are one of the most commonly used embolic agents for bronchial artery embolization (BAE) in patients with hemoptysis [1,2]. However, little evidence is available on their efficacy and safety during extracorporeal membrane oxygenation (ECMO), and with appropriate anticoagulation management. The homeostatic mechanism of metallic coils depends on the ability of the patient to form a thrombus that fills the microscopic gap between the coils; therefore, continuous anticoagulation during ECMO can hinder complete vessel occlusion. We herein report a case of successful BAE using hydrogel coils for hemoptysis during ECMO.

## Case report

**Fig. 1:**
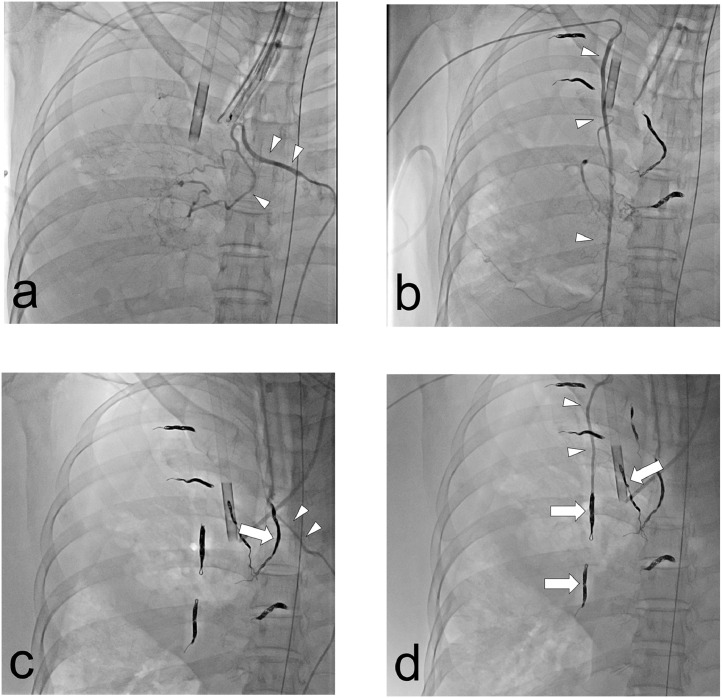
Angiographic findings. (A, B) Right bronchial artery (intercostobronchial trunk) and right internal mammary artery before coil embolization on day 5 (arrowheads). (C, D) The same arteries on day 13 (arrowheads); there was no recanalization found on the coils that had been placed on day 5 (arrows).

A 58-year-old woman with bronchiectasis was admitted to the emergency department with massive hemoptysis and severe respiratory failure, which required intubation and admission to the intensive care unit. Three days before admission, she had been hospitalized at another hospital, where she developed hemoptysis caused by bronchoscopy with brush cytology at the right B4b; she was discharged after temporary cessation of the bleeding without any intervention. Chest computed tomography (CT) showed bronchiectasis in the right middle lobe and diffused infiltration in both lungs. Contrast-enhanced CT revealed enlargement of 2 right bronchial arteries and several nonbronchial systemic arteries. Laboratory tests demonstrated no decrease in platelets or coagulation disorders. On day 3, veno-venous ECMO was initiated due to uncontrollable hypoxemia and hypercapnia. A heparin-coated ECMO circuit and cannula were used, and intravenous unfractionated heparin was continuously administered, except during the initiation phase, to maintain an activated clotting time of 150-180 seconds. The activated clotting time reagent and analyzer were C-ACT (celite) and Actalyke MINI II (TRYTECH Co., Ltd., Tokyo, Japan), respectively (normal range, 105-130 seconds; blood sample volume, 2.0 ml). Platelet concentrate was transfused to maintain a platelet count of 50,000/µl. Red blood cells were transfused to maintain a hemoglobin level of 10 g/dl. Fresh frozen plasma was transfused to maintain a fibrinogen level of 150 mg/dl. Only gelatin sponge particles and metallic coils were covered by the medical insurance in the authors’ country; therefore, the first BAE using gelatin sponge particles was performed on day 3, resulting in re-hemoptysis. On day 5, the second BAE was performed using metallic coils (Figs. 1A and B). Hydrogel coils, in addition to normal bare platinum coils, were intentionally used to avoid incomplete vessel occlusion caused by heparin. However, on day 6, active bleeding from the right middle lobe was still observed after the removal of the endobronchial clot with a bronchoscope. A repeat contrast-enhanced CT revealed an untreated ectopic bronchial artery arising from the right subclavian artery. On day 13, the third BAE using coils was performed; at this time, no recanalization of the arteries that had been embolized at the second BAE was observed (Figs. 1C and D). Following the third BAE, no bleeding was observed. On day 18, ECMO and intravenous heparin were stopped. A summary of these three BAEs is shown in Table 1. Mechanical ventilation was stopped on day 25. On day 60, follow-up contrast-enhanced CT revealed no recanalization of any arteries embolized with coils. On day 76, the patient was deemed fully recovered and discharged without any sequelae. There had been no recurrence of bleeding post-BAE at the time of this report (at least 6 months).

**Table 1:** Summary of bronchial artery embolization (BAE)

	Embolized artery	Coils placed
First BAE (day 3)	Right BA (common bronchial trunk)	(Gelatin sponge particles)^a^
Second BAE (day 5)	Right BA (common bronchial trunk)	2 HGCs + 3 BPCs^b^
	Right BA (intercostobronchial trunk)	1 HGC + 4 BPCs^b^
	Right fourth intercostal artery	1 HGC + 2 BPCs^b^
	Right fifth intercostal artery	1 HGC + 2 BPCs^b^
	Ectopic BA (right internal mammary artery)	1 HGC + 3 BPCs^b^
	Right internal mammary artery	1 HGC + 2 BPCs (proximal part)^b^ 2 BPCs (distal part)^b^
Third BAE (day 13)	Ectopic BA (right subclavian artery)	2 HGCs + 2 BPCs
	Right lateral thoracic artery	1 HGC + 2 BPCs
	Right fourth intercostal artery^c^	1 HGC + 1 BPC

BA, bronchial artery; BAE, bronchial artery embolization; BPC, bare platinum coil (C-STOPPER; PIOLAX MEDICAL DEVICES Inc., Yokohama, Japan); HGC, hydrogel-coated coil (AZUR; Terumo Corp., Tokyo, Japan).

a Selective angiography on day 5 showed recanalization of the right bronchial artery, which had been embolized using gelatin sponge particles.

b Selective angiography on day 13 showed no recanalization of any of the arteries that had been embolized using coils.

c Two coils were added to the right fourth intercostal artery because selective angiography on day 13 revealed a collateral branch proximal to the coils that had been placed on day 5.

## Discussion

Although research regarding the suitable choice of embolic agents during ECMO is lacking, this case revealed that BAE using coils was effective and safe during ECMO with continuous anticoagulation using intravenous unfractionated heparin. In this regard, the use of hydrogel coils can be considered an important factor in the success of the present case, since they provide a greater volume occlusion independent of acute thrombus than bare platinum coils [3]. A 2020 randomized controlled trial [4] reported that coiling of intracranial aneurysms with hydrogel coils resulted in less recurrence compared to bare platinum coils. In addition, coils are easy to use and safe. A 2021 nationwide observational study in Japan [5] revealed that patients who underwent BAE using coils had a lower prevalence of spinal cord infarction, which is the most serious complication of BAE, than patients who underwent BAE with gelatin sponge particles or N-butyl-2-cyanoacrylate. However, a further study with a larger sample size is needed to verify whether hydrogel coils are more appropriate than other embolic agents in patients with hemoptysis on ECMO.

ECMO is used in the most severe respiratory failure cases of hemoptysis to allow the respiratory system to recover or as a bridge to other interventions. However, ECMO in patients with hemoptysis is challenging because it requires simultaneous anticoagulation. Anticoagulation therapy can exacerbate hemoptysis, but thrombosis formation, either systemic or within the ECMO circuit, can also be life-threatening [6]. Little evidence is available regarding the adequate dosing and monitoring of anticoagulants in patients with hemoptysis on ECMO; moreover, adequate anticoagulation management for successful coil embolization is also unknown. Therefore, this case presents a successful example of this type of management.

In conclusion, although continuous anticoagulation during ECMO might hinder complete vessel occlusion by metallic coils or induce early recanalization, our experience showed that BAE using hydrogel coils was effective and safe. Additionally, the case presents a successful example of anticoagulation management for patients with hemoptysis on ECMO who undergo BAE using coils.

## Patient consent

The authors declare that appropriate written informed consent was obtained for the publication of this manuscript and accompanying images.
